# Supplementary material for: Worldwide genetic diversity of Plasmodium vivax Pv47 is consistent with natural selection by anopheline mosquitoes
Source: Nat Commun. 2025 Aug 9;16:7363. doi: 10.1038/s41467-025-62680-3 (PMC12335571; doi:10.1038/s41467-025-62680-3)
Supplement: Supplementary file 1 — Supplementary Information [file 41467_2025_62680_MOESM1_ESM.pdf]

Supplementary Information

Worldwide genetic diversity of *Plasmodium* vivax *Pv47* is consistent with natural selection by anopheline mosquitoes

Alvaro Molina-Cruz, Lilia Gonzalez-Ceron, Ankit Dwivedi, Tran Zen B. Torres, Nadia Raytselis, Micah Young, Nitin Kamath, Colton McNinch, Xinzhuan Su, Anthony Ford, Marcelo U. Ferreira, Myriam Arévalo-Herrera, Sócrates Herrera, Eugenia Lo, Joana C. Silva and Carolina Barillas-Mury.

Table S1. Pv47 sequences by country of origin.

Country	No. Samples	Haplotypes
El Salvador	1	1
Mexico	81	9
Colombia	90	10
Peru	69	16
Brazil	83	24
Mauritania	3	2
Ethiopia	211	21
Sudan	23	6
Madagascar	6	3
Iran	7	4
Afghanistan	54	16
India	26	6
Bangladesh	11	9
China	9	6
North Korea	1	1
Korea	4	4
Philippines	5	5
Thailand	118	51
Cambodia	112	37
Vietnam	66	33
Laos	1	1
Bhutan	3	3
Myanmar	7	6
Indonesia	3	2
Malaysia	60	9
Papua Indonesia	120	32
Papua New Guinea (PNG)	19	8
Vanuatu	6	6
Total samples	1199	

Table S2. Single nucleotide polymorphisms (SNP) in Pv47 gene sequence.

SNP Number	SNP Location (bp)	Reference Allele (SalI)	Alternate allele	Mutation type	AA* substitution
1	16	T	G	NS	F6V
2	62	G	A	NS	S21N
3	66	C	A	NS	F22L
4	70	T	C	NS	F24L
5	78	G	A	S	T26T
6	79	A	G	NS	K27E
7	80	A	G	NS	K27R
8	85	T	A	NS	L29I
9	91	G	A	NS	D31N
10	170	G	C	NS	S57T
11	185	G	A	NS	S62N
12	226	G	A	NS	G76K
13	227	G	A	NS	G76E
14	228	A	G	NS	G76K
15	241	G	C	NS	E81Q
16	244	T	G	NS	L82V
17	255	G	A	S	V85V
18	307	A	C	NS	N103H
19	336	A	T	NS	L112F
20	337	G	A	NS	D113N
21	360	A	C	NS	R120S
22	385	G	T	NS	V129L
23	390	G	A	S	L130L
24	391	G	A	NS	V131I
25	408	T	C	S	Y136Y
26	412	C	T	NS	H138Y
27	453	G	C	S	T151T
28	460	G	T	S	G154G
29	462	G	A	S	G154G
30	466	G	A	NS	D156G
31	467	A	G	NS	D156S
32	535	C	A	NS	P179T
33	578	C	T	NS	T193I
34	581	A	G	NS	D194G
35	597	A	G	S	G199G
36	664	C	T	NS	L222F
37	688	G	A	NS	V230I
38	699	G	T	NS	M233I
39	709	T	A	NS	F237I
40	720	G	T	NS	E240D
41	724	G	T	NS	V242F
42	758	C	T	NS	S253F
43	759	C	T	S	S253S
44	773	C	T	NS	T258I
45	785	T	A/C	NS	I262T/K
46	787	G	A	NS	E263K
47	791	G	A	NS	S264N
48	817	A	G	NS	I273M
49	819	A	G	NS	I273V
50	849	C	T	S	G283G
51	966	T	C	S	I322I
52	984	C	T	S	C328C
53	1010	G	T	NS	C337F
54	1020	C	T	S	H340H
55	1021	G	A	NS	V341I
56	1023	C	T	S	V341V
57	1024	C	T	S	L342L
58	1050	G	A	S	V350V
59	1056	C	T	S	S352S
60	1061	A	T	NS	H354L
61	1068	C	T	S	F356F
62	1077	C	A	S	T359T
63	1107	C	T	S	F369F
64	1116	T	C	S	N372N
65	1118	C	T	NS	A373V
66	1143	G	A	S	G381G
67	1182	C	T	S	C394C
68	1189	G	A	NS	E397K
69	1260	C	T	S	L420L
70	1282	G	A	NS	A428T
71	1302	G	A	NS	Stop

*AA Amino acid substitution.

Table S3. Polymorphisms and evolution of Pv47 gene coding sequences worldwide.

	Segregating sites (S)	Haplotypes (H)	Haplotype diversity (Hd)	π[a](SE)	dS[b]	dN[c]	dS-dN(SE)	p (Z stat)[d]	Tajima's D (p-value)[e]
**All Regions (N=1199)**	71	209	0.950	0.0040 (0.0010)	0.0020	0.0045	-0.0025 (0.0020)	0.1771 (1.3576)	-1.1925 (0.2202)
**Sigpep[f] (1-63)**	2	4	0.040	0.0007 (0.0005)	0.0000	0.0009	-0.0009 (0.0007)	0.1477 (1.4573)	-0.9127 (0.3761)
**D1 (64-540)**	30	54	0.738	0.0045 (0.0016)	0.0044	0.0045	-0.0001 (0.0044)	0.9747 (0.0318)	-1.1165 (0.2583)
**D2 (541-837)**	17	49	0.874	0.0081 (0.0032)	0.0007	0.0099	-0.0092 (0.0042)	**0.0302** (2.1940)	0.1630 (0.8157)
**D3 (838-1230)**	19	22	0.534	0.0015 (0.0012)	0.0011	0.0016	-0.0006 (0.0017)	0.7372 (0.3363)	-1.7347 (**0.0455**)
**GPI[g] (1231-1299)**	2	3	0.003	0.0000 (0.0000)	0.0001	0.0000	0.0001 (0.0001)	0.6033 (-0.5211)	-1.0783 (0.2787)
**Mexico (N=81)**	8	9	0.527	0.0006 (0.0004)	0.0001	0.0008	-0.0007 (0.0005)	0.1564 (1.4264)	-1.2662 (0.2015)
**Sigpep (1-63)**	0	1	0.000	0.0000 (0.0000)	0.0000	0.0000	0.0000 (0.0000)	1.0000 (0.0000)	N/A
**D1 (64-540)**	4	5	0.473	0.0015 (0.0010)	0.0003	0.0018	-0.0015 (0.0013)	0.2203 (1.2322)	-0.2730 (0.8291)
**D2 (541-837)**	1	2	0.025	0.0001 (0.0001)	0.0000	0.0001	-0.0001 (0.0001)	0.3373 (0.9633)	-1.0513 (0.3039)
**D3 (838-1230)**	2	3	0.049	0.0001 (0.0001)	0.0000	0.0002	-0.0002 (0.0001)	0.1615 (1.4087)	-1.4060 (0.1477)
**GPI (1231-1299)**	1	2	0.025	0.0004 (0.0004)	0.0000	0.0005	-0.0005 (0.0005)	0.3285 (0.9810)	-1.0513 (0.3039)
**South America (N=243)**	36	40	0.468	0.0009 (0.0002)	0.0007	0.0010	-0.0002 (0.0005)	0.6627 (0.4373)	**-2.2668** (**0.0042**)
**Sigpep (1-63)**	2	3	0.041	0.0007 (0.0005)	0.0000	0.0009	-0.0009 (0.0006)	0.1682 (1.3863)	-1.1615 (0.2424)
**D1 (64-540)**	19	17	0.307	0.0016 (0.0005)	0.0015	0.0017	-0.0002 (0.0013)	0.8696 (0.1645)	-1.9613 (**0.0221**)
**D2 (541-837)**	9	11	0.194	0.0009 (0.0004)	0.0001	0.0011	-0.0009 (0.0005)	0.0657 (1.8572)	-1.8351 (**0.0372**)
**D3 (838-1230)**	5	6	0.104	0.0003 (0.0001)	0.0006	0.0002	0.0005 (0.0004)	0.2300 (-1.2068)	-1.6289 (0.0757)
**GPI (1231-1299)**	0	1	0.000	0.0000 (0.0000)	0.0000	0.0000	0.0000 (0.0000)	1.0000 (0.0000)	N/A
**Africa (N=243)**	30	25	0.783	0.0015 (0.0007)	0.0002	0.0019	-0.0016 (0.0008)	0.0403 (2.0728)	-1.6668 (0.0672)
**Sigpep (1-63)**	1	2	0.008	0.0001 (0.0001)	0.0000	0.0002	-0.0002 (0.0000)	0.3381 (0.9618)	-0.9319 (0.3672)
**D1 (64-540)**	10	5	0.147	0.0004 (0.0003)	0.0002	0.0005	-0.0003 (0.0004)	0.0884 (1.7178)	**-2.0073** (**0.0180**)
**D2 (541-837)**	11	13	0.629	0.0041 (0.0024)	0.0000	0.0051	-0.0051 (0.0029)	0.0712 (1.8202)	-0.7809 (0.4628)
**D3 (838-1230)**	7	5	0.514	0.0014 (0.0013)	0.0006	0.0017	-0.0011 (0.0018)	0.5288 (0.6316)	-1.0695 (0.2892)
**GPI (1231-1299)**	0	1	0.000	0.0000 (0.0000)	0.0000	0.0000	0.0000 (0.0000)	1.0000 (0.0000)	N/A
**Middle East / S Asia (N=98)**	40	29	0.707	0.0017 (0.0004)	0.0019	0.0015	0.0005 (0.0010)	0.6580 (-0.4438)	**-2.2537** (**0.0059**)
**Sigpep (1-63)**	1	2	0.040	0.0006 (0.0006)	0.0000	0.0009	-0.0009 (0.0008)	0.2968 (1.0479)	-0.9114 (0.3819)
**D1 (64-540)**	18	12	0.460	0.0020 (0.0007)	0.0010	0.0020	-0.0009 (0.0010)	0.3862 (0.8696)	**-2.0725** (**0.0156**)
**D2 (541-837)**	10	9	0.313	0.0022 (0.0010)	0.0000	0.0027	-0.0027 (0.0012)	**0.0419** (2.0570)	-1.7255 (0.0603)
**D3 (838-1215)**	10	10	0.374	0.0395 (0.0079)	0.0486	0.0358	0.0128 (0.0105)	0.2407 (-1.1790)	**-2.9951** (**0.0000**)
**GPI (1216-1299)**	0	1	0.000	0.0000 (0.0000)	0.0000	0.0000	0.0000 (0.0000)	1.0000 (0.0000)	N/A
**E Asia / SE Asia (N=389)**	51	100	0.942	0.0026 (0.0009)	0.0006	0.0031	-0.0025 (0.0011)	**0.0253** (2.2657)	-1.6056 (0.0785)
**Sigpep (1-63)**	2	3	0.026	0.0004 (0.0003)	0.0000	0.0006	-0.0006 (0.0004)	0.1461 (1.4630)	-1.1386 (0.2514)
**D1 (64-540)**	24	21	0.556	0.0016 (0.0009)	0.0006	0.0019	-0.0013 (0.0011)	0.2351 (1.1933)	**-2.0343** (**0.0144**)
**D2 (541-837)**	15	30	0.813	0.0068 (0.0029)	0.0004	0.0084	-0.0080 (0.0037)	**0.0389** (2.0884)	-0.3125 (0.8092)
**D3 (838-1215)**	8	12	0.488	0.0014 (0.0011)	0.0009	0.0015	-0.0006 (0.0015)	0.6656 (0.4333)	-1.1546 (0.2435)
**GPI (1216-1299)**	1	2	0.005	0.0001 (0.0001)	0.0003	0.0000	0.0003 (0.0003)	0.3662 (-0.9070)	-0.8855 (0.3948)
**PI/PNG/Van (N=145)**	35	43	0.930	0.0035 (0.0009)	0.0009	0.0042	-0.0033 (0.0012)	0.0106 (2.5841)	-0.8391 (0.4255)
**Sigpep (1-63)**	2	3	0.026	0.0027 (0.0024)	0.0000	0.0037	-0.0037 (0.0031)	0.2241 (1.2220)	-0.7653 (0.4733)
**D1 (64-540)**	15	9	0.698	0.0025 (0.0012)	0.0006	0.0030	-0.0024 (0.0018)	0.1898 (1.3185)	-1.4870 (0.1172)
**D2 (541-837)**	15	18	0.755	0.0096 (0.0035)	0.0021	0.0115	-0.0094 (0.0042)	**0.0224** (2.3133)	0.1185 (0.8669)
**D3 (838-1230)**	3	4	0.336	0.0009 (0.0007)	0.0009	0.0009	0.0000 (0.0012)	0.9787 (-0.0268)	-0.5929 (0.5927)
**GPI (1231-1299)**	0	1	0.000	0.0000 (0.0000)	0.0000	0.0000	0.0000 (0.0000)	1.0000 (0.0000)	N/A

[a]π, Nucleotide diversity.

[b]dS, average number of synonimous polymorphisms between two sequences.

[c]dN, average number of non-synonymous polymorphisms between two sequences.

[d]Z-Test, two-sided.

[e]Tajima's D test, two-sided.

[f]Sigpep, Predicted signal peptide sequence.

[g]GPI, Sequence after a predicted ω-cleavage site.

Table S4. Polymorphisms and evolution of Pfs47 gene coding sequences worldwide.

	Segregating sites (S)	Haplotypes (H)	Haplotype diversity (Hd)	π[a](SE)	dS[b]	dN[c]	dS-dN(SE)	p (Z stat)[d]	Tajima's D (p-value)[e]
**All Regions (N=4971)**	83	209	0.89	0.0031 (0.0009)	0.0002	0.0036	-0.0034 (0.0012)	**0.0040** (2.9686)	-1.4122 (0.1222)
**Sigpep[f] (1-78)**	6	8	0	0.0000 (0.0000)	0.0001	0.0000	0.0000 (0.0001)	0.8190 (-0.2295)	-1.4885 (0.0972)
**D1 (79-549)**	22	24	0.66	0.0021 (0.0012)	0.0000	0.0026	-0.0026 (0.0016)	0.0980 (1.6610)	-1.2667 (0.1800)
**D2 (550-846)**	21	72	0.78	0.0098 (0.0035)	0.0000	0.0120	-0.0120 (0.0045)	**0.0070** (2.7410)	0.5160 (0.5688)
**D3 (847-1239)**	26	29	0.13	0.0003 (0.0003)	0.0001	0.0004	-0.0003 (0.0004)	0.3300 (0.9666)	-2.1193 (**0.0046**)
**GPI[g] (1239-1317)**	8	10	0.09	0.0012 (0.0008)	0.0034	0.0005	0.0029 (0.0037)	0.4260 (-0.7981)	-1.4701 (0.1029)
**South America (N=121)**	7	4	0.52	0.0006 (0.0004)	0.0000	0.0008	-0.0008 (0.0004)	0.1475 (1.4578)	-0.8015 (0.4498)
**Sigpep (1-78)**	0	1	0	0.0000 (0.0000)	0.0000	0.0000	0.0000 (0.0000)	1.0000 (0.0000)	N/A
**D1 (79-549)**	2	3	0.51	0.0016 (0.0011)	0.0000	0.0021	-0.0021 (0.0014)	0.1139 (1.5925)	1.5529 (0.1401)
**D2 (550-846)**	5	2	0.02	0.0003 (0.0001)	0.0000	0.0004	-0.0004 (0.0002)	0.0733 (1.8069)	-1.8814 (**0.0341**)
**D3 (847-1239)**	0	1	0	0.0000 (0.0000)	0.0000	0.0000	0.0000 (0.0000)	1.0000 (0.0000)	N/A
**GPI (1239-1317)**	0	1	0	0.0000 (0.0000)	0.0000	0.0000	0.0000 (0.0000)	1.0000 (0.0000)	N/A
**Africa (N=3126)**	72	158	0.81	0.0013 (0.0004)	0.0001	0.0015	-0.0014 (0.0007)	0.1550 (1.4320)	-2.0507 (0.0083)
**Sigpep (1-78)**	6	7	0.01	0.0001 (0.0000)	0.0001	0.0001	0.0000 (0.0001)	1.0000 (0.0000)	-1.5348 (0.0858)
**D1 (79-549)**	19	20	0.37	0.0008 (0.0007)	0.0000	0.0009	-0.0009 (0.0010)	0.1930 (1.3102)	-1.7711 (**0.0356**)
**D2 (550-846)**	21	57	0.63	0.0039 (0.0019)	0.0000	0.0048	-0.0048 (0.0020)	**0.0300** (2.2400)	-1.1528 (0.2361)
**D3 (847-1239)**	21	26	0.19	0.0005 (0.0004)	0.0000	0.0005	-0.0005 (0.0005)	1.0000 (0.0000)	-2.0122 (**0.0105**)
**GPI (1239-1317)**	6	7	0.02	0.0002 (0.0002)	0.0015	0.0000	0.0015 (0.0007)	1.0000 (0.0000)	-1.5136 (0.0918)
**S Asia (Banglandesh; N = 80)**	10	18	0.87	0.0020 (0.0007)	0.0000	0.0025	-0.0025 (0.0008)	**0.0103** (2.6065)	0.7469 (0.4585)
**Sigpep (1-78)**	0	1	0	0.0000 (0.0000)	0.0000	0.0000	0.0000 (0.0000)	1.0000 (0.0000)	N/A
**D1 (79-549)**	4	6	0.60	0.0016 (0.0010)	0.0000	0.0021	-0.0021 (0.0016)	0.1310 (1.5190)	-0.1189 (0.9472)
**D2 (550-846)**	5	8	0.56	0.0049 (0.0021)	0.0000	0.0060	-0.0060 (0.0027)	**0.0375** (2.1038)	0.9628 (0.3481)
**D3 (847-1239)**	0	0	0	0.0000 (0.0000)	0.0000	0.0000	0.0000 (0.0000)	1.0000 (0.0000)	N/A
**GPI (1239-1317)**	1	2	0.38	0.0048 (0.0053)	0.0000	0.0064	-0.0064 (0.0060)	0.2849 (1.0741)	1.0583 (0.3052)
**SE Asia (N = 1595)**	21	44	0.69	0.0011 (0.0004)	0.0007	0.0012	-0.0005 (0.0009)	0.6022 (0.5226)	-1.0320 (0.3037)
**Sigpep (1-78)**	0	1	0	0.0000 (0.0000)	0.0000	0.0000	0.0000 (0.0000)	1.0000 (0.0000)	N/A
**D1 (79-549)**	3	5	0.52	0.0011 (0.0091)	0.0001	0.0014	-0.0013 (0.0010)	0.4165 (0.8153)	0.5201 (0.5718)
**D2 (550-846)**	10	15	0.33	0.0022 (0.0012)	0.0000	0.0027	-0.0027 (0.0015)	0.1090 (1.6122)	-0.9030 (0.3819)
**D3 (847-1239)**	5	4	0.01	0.0000 (0.0000)	0.0001	0.0000	0.0001 (0.0001)	0.3270 (0.9842)	-1.4949 (0.1006)
**GPI (1239-1317)**	4	7	0.22	0.0030 (0.0020)	0.0096	0.0011	0.0085 (0.0084)	0.5083 (0.6635)	-0.4831 (0.6834)
**PNG (N = 59)**	12	8	0.64	0.0022 (0.0008)	0.0000	0.0028	-0.0028 (0.0010)	**0.0070** (2.7397)	0.2317 (0.7958)
**Sigpep (1-78)**	0	1	0	0.0000 (0.0000)	0.0000	0.0000	0.0000 (0.0000)	1.0000 (0.0000)	N/A
**D1 (79-549)**	2	3	0.47	0.0018 (0.0013)	0.0000	0.0023	-0.0023 (0.0017)	0.1790 (1.3529)	1.6672 (0.1114)
**D2 (550-846)**	10	7	0.63	0.0069 (0.0029)	0.0000	0.0085	-0.0085 (0.0034)	**0.0017** (2.4220)	-0.2649 (0.8322)
**D3 (847-1239)**	0	1	0	0.0000 (0.0000)	0.0000	0.0000	0.0000 (0.0000)	1.0000 (0.0000)	N/A
**GPI (1239-1317)**	0	1	0	0.0000 (0.0000)	0.0000	0.0000	0.0000 (0.0000)	1.0000 (0.0000)	N/A

[a]π, Nucleotide diversity.
[b]dS, average number of synonimous polymorphisms between two sequences.
[c]dN, average number of non-synonymous polymorphisms between two sequences.
[d]Z-Test, two-sided.
[e]Tajima's D test, two-sided.
[f]Sigpep, Predicted signal peptide sequence.
[g]GPI, Sequence after a predicted ω-cleavage site.

	Guinea	Mali	Senegal	The_Gambia	Burkina	Ghana	Uganda	Kenya	Tanzania	Malawi	Madagascar	DRC	Brazil	French_Guiana	Peru	Colombia	Bangladesh	Cambodia	Laos	Myanmar	Thailand	Vietnam
Guinea																						
Mali	0																					
Senegal	0.01	0.01																				
The_Gambia	0.01	0.01	0.01																			
Burkina	0.06	0.07	0.11	0.06																		
Ghana	0	0	0.01	0.02	0.09																	
Uganda	0.11	0.14	0.15	0.07	0.13	0.14																
Kenya	0.16	0.18	0.18	0.12	0.21	0.18	0															
Tanzania	0.11	0.13	0.15	0.07	0.11	0.14	0	0.02														
Malawi	0.27	0.29	0.29	0.23	0.35	0.28	0.08	0.02	0.10													
Madagascar	0.30	0.32	0.32	0.26	0.38	0.31	0.12	0.05	0.15	0.02												
DRC	0.11	0.11	0.14	0.12	0.20	0.09	0.14	0.16	0.15	0.22	0.25											
Brazil	0.83	0.82	0.82	0.84	0.93	0.81	0.89	0.85	0.87	0.87	0.86	0.79										
French_Guiana	0.74	0.74	0.73	0.75	0.82	0.73	0.80	0.77	0.79	0.79	0.78	0.72	0.28									
Peru	0.76	0.75	0.75	0.76	0.84	0.74	0.81	0.78	0.80	0.80	0.79	0.73	0.23	0								
Colombia	0.86	0.86	0.86	0.87	0.94	0.85	0.91	0.88	0.90	0.89	0.88	0.82	1.00	0.51	0.56							
Bangladesh	0.46	0.45	0.44	0.47	0.56	0.44	0.54	0.52	0.53	0.55	0.54	0.48	0.65	0.58	0.60	0.73						
Cambodia	0.78	0.78	0.78	0.79	0.85	0.77	0.82	0.80	0.82	0.81	0.79	0.76	0.89	0.83	0.84	0.91	0.49					
Laos	0.75	0.75	0.75	0.76	0.81	0.74	0.79	0.77	0.78	0.78	0.76	0.73	0.85	0.79	0.80	0.87	0.45	0.03				
Myanmar	0.78	0.78	0.78	0.79	0.85	0.77	0.83	0.80	0.82	0.82	0.79	0.76	0.90	0.83	0.84	0.91	0.47	0.04	0.09			
Thailand	0.82	0.82	0.82	0.83	0.88	0.81	0.86	0.83	0.85	0.85	0.83	0.79	0.93	0.86	0.87	0.94	0.53	0.04	0.13	0.02		
Vietnam	0.78	0.77	0.77	0.78	0.83	0.76	0.82	0.79	0.81	0.80	0.78	0.75	0.88	0.82	0.83	0.89	0.49	0.04	0.01	0.08	0.13	
PNG	0.43	0.43	0.42	0.44	0.53	0.42	0.51	0.49	0.51	0.53	0.51	0.45	0.63	0.56	0.58	0.70	0.03	0.42	0.38	0.40	0.46	0.42

Table S5. Population structure of Pfs47 haplotypes. The fixation index (F_{ST}) between Pfs47 country populations analyzed is shown.

Table S6. Infectivity of in blood samples from Southern Mexico to local vectors *Anopheles albimanus* and *Anopheles pseudopunctipennis*. Laboratory colonies of An. albimanus and An. pseudopunctipennis were feed simultaneously with blood samples and the infection prevalence and average number of oocysts per experiment were determined. N, Number of mosquitoes analyzed for infectivity in the midgut. Inf. Prev., Infection Prevalence; SD, Standard Deviation; Chi-Square, two-sided; *, p< 0.05; **, p < 0.01; ***, p < 0.001; ****, p < 0.0001.

			Infectivity to *An. albimanus*				Infectivity to *An. pseudopuctipennis*				
**Sample**	**Subpopulation#**	**Preferential vector**	**n**	**Inf. Prev.**	**Aver. Oocyst**	**SD**	**n**	**Inf. Prev.**	**Aver. Oocyst**	**SD**	**Prevalence difference *An. alb*. vs *An. Pseudo*. (p,significance)†**
MEX-009	C1	*An. alb.*	12	0.7	16.8	6.1	8	0.0	0.0	0.0	0.0014**
MEX-036	C1	"	25	0.6	45.1	32.8	6	0.0	0.0	0.0	0.0177*
MEX-051	C1	"	25	0.6	14.1	13.8	10	0.0	0.0	0.0	0.0016**
MEX-053	C1	"	25	0.7	48.5	32.1	10	0.3	3.0	2.6	0.0196*
MEX-054	C1	"	25	0.7	33.5	16.3	10	0.0	0.0	0.0	0.0001***
MEX-055	C1	"	25	0.8	55.7	36.4	11	0.0	0.0	0.0	<0.0001****
MEX-059	C1	"	25	0.2	23.6	22.1	10	0.0	0.0	0.0	0.0363*
MEX-060	C1	"	25	0.4	51.4	45.9	11	0.0	0.0	0.0	0.0160*
MEX-061	C1	"	25	0.9	73.6	47.4	23	0.0	0.0	0.0	<0.0001****
MEX-063	C1	"	25	0.6	14.8	10.3	20	0.1	2.0	1.0	0.0007***
MEX-065	C1	"	25	0.4	12.8	12.4	11	0.0	0.0	0.0	0.0160*
MEX-136	C1	"	10	0.8	18.0	14.0	8	0.0	0.0	0.0	0.0011**
MEX-158	C1	"	10	0.8	86.9	59.8	20	0.0	0.0	4.9	<0.0001****
MEX-223	C1	"	25	0.8	55.4	44.1	6	0.1	1.0	0.0	0.0075**
MEX-255	C1	"	25	0.9	24.2	19.4	10	0.0	0.0	0.0	<0.0001****
MEX-049	F2	*An. pseudo.*	25	0.0	1.0	0.0	6	0.8	14.6	16.3	0.0002***
MEX-056	F1	"	25	0.1	1.3	0.5	7	1.0	41.5	28.0	<0.0001****
MEX-062	F2	"	25	0.0	0.0	0.0	7	1.0	34.7	17.1	<0.0001****
MEX-098	F2	"	25	0.0	0.0	0.0	7	0.4	25.0	19.0	0.0071**
MEX-112	F2	"	25	0.2	4.0	3.6	20	0.9	41.0	25.0	<0.0001****
MEX-120	F2	"	25	0.0	0.0	0.0	6	0.5	47.0	64.0	0.0044**
MEX-122	F1	"	25	0.0	0.0	0.0	10	0.5	13.0	9.0	0.0008***
MEX-141	F2	"	10	0.0	0.0	0.0	5	0.8	23.0	17.0	0.0037**
MEX-144	F2	"	25	0.0	3.0	0.0	10	0.7	32.0	27.0	0.0001***
MEX-145	F2	"	9	0.0	0.0	0.0	10	0.5	6.8	3.3	0.0325*
MEX-178	F2	"	25	0.0	0.0	0.0	7	0.7	6.2	2.7	0.0001***
MEX-179	F2	"	25	0.0	0.0	0.0	11	0.6	7.8	7.3	<0.0001****
MEX-199	F2	"	25	0.0	1.0	0.0	10	0.8	70.0	52.1	<0.0001****
MEX-201	F1	"	25	0.0	0.0	0.0	7	1.0	54.5	18.9	<0.0001****
MEX-202	F2	"	25	0.0	0.0	0.0	10	0.8	14.0	10.0	<0.0001****
MEX-208	F1	"	25	0.0	0.0	0.0	10	0.9	20.3	10.9	<0.0001****
MEX-209	F1	"	25	0.1	3.7	3.5	10	1.0	48.6	30.2	<0.0001****
MEX-211	F2	"	25	0.0	0.0	0.0	7	0.7	13.8	19.9	0.0001***
MEX-214	F2	"	25	0.0	0.0	0.0	10	0.5	22.6	13.3	0.0008***
MEX-216	F2	"	25	0.1	7.5	0.7	10	0.9	114.2	43.6	<0.0001****
MEX-218	F1	"	25	0.0	0.0	0.0	8	1.0	25.5	19.6	<0.0001****
MEX-235	F2	"	15	0.1	1.0	0.0	10	0.6	11.0	8.0	0.0068**
MEX-241	F1	"	9	0.0	0.0	0.0	10	0.6	2.2	1.3	0.0108*
MEX-248	F2	"	10	0.0	0.0	0.0	5	1.0	31.6	26.6	0.0003***
MEX-249	F2	"	25	0.0	6.0	0.0	10	0.6	52.0	32.5	0.0008***
MEX-251	F2	"	25	0.0	0.0	0.0	5	1.0	7.0	4.1	<0.0001****
MEX-253	F2	"	25	0.0	0.0	0.0	10	0.7	27.0	20.7	<0.0001****
MEX-254	F1	"	25	0.0	5.0	0.0	10	0.5	40.8	24.0	0.0040**

#Population structure according to Joy et al. (14).

† Chi-Squared test.

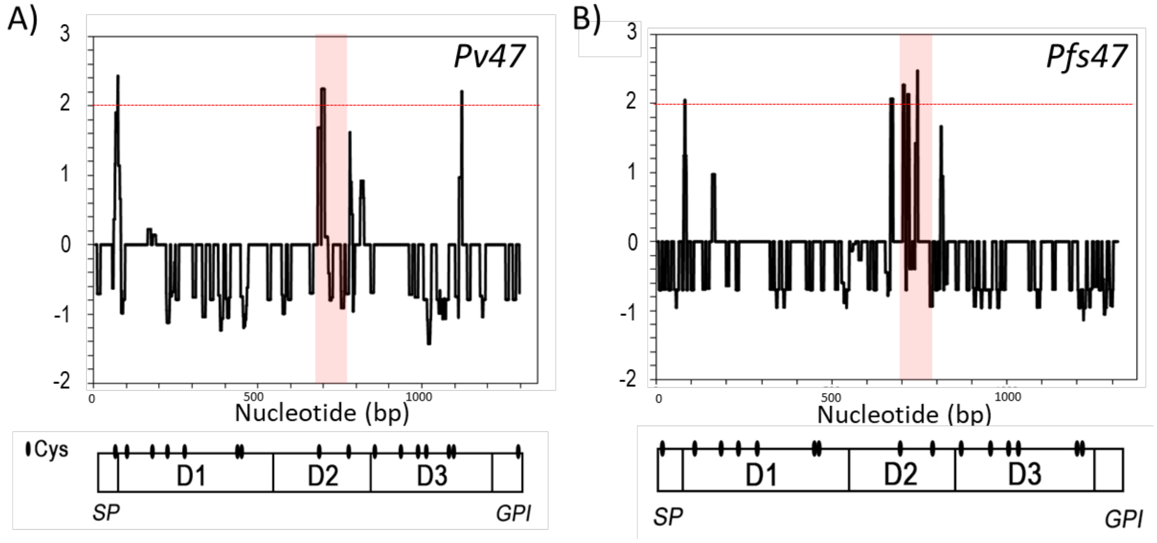

Figure S1. Signatures of selection in Pv47 and Pfs47 sequences. Tajima's D per 15 nt window is shown for Pv47 (A) and Pfs47 (B). The predicted protein domain structure and Cys location are indicated for both Pv47 and Pfs47. SP, signal peptide; D1, domain 1; D2, domain 2; D3, Domain 3; GPI, predicted glycosilphosphatidyl inositol anchoring. The region between two Cys in D2 is shaded in red.

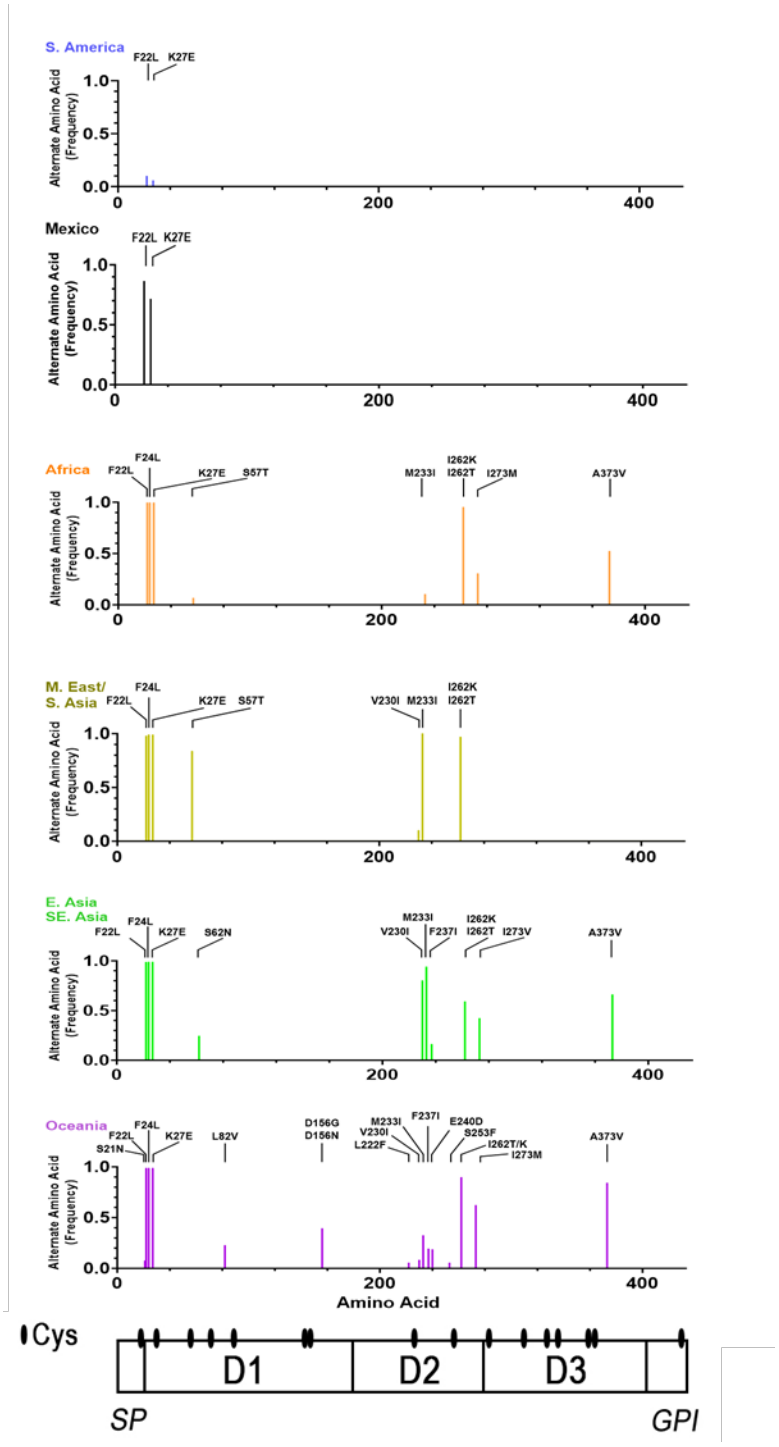

Fig S2. Main amino acid polymorphisms in Pv47 protein sequences. The alternate amino acid frequencies for polymorphisms that have >0.05 frequency in any of the geographic region analyzed are indicated. The domain structure of Pv47 is indicated (bottom), with the relative position of Cys. SP, signal peptide; D1, domain 1; D2, domain 2; D3, Domain 3; GPI, predicted glycosilphosphatidyl inositol anchoring; S. America, South America; M.

East, Middle East; S. Asia, South Asia; E. Asia, East Asia; SE Asia, Southeast Asia; Oceania, Papua New Guinea, Papua Indonesia, and Vanuatu.

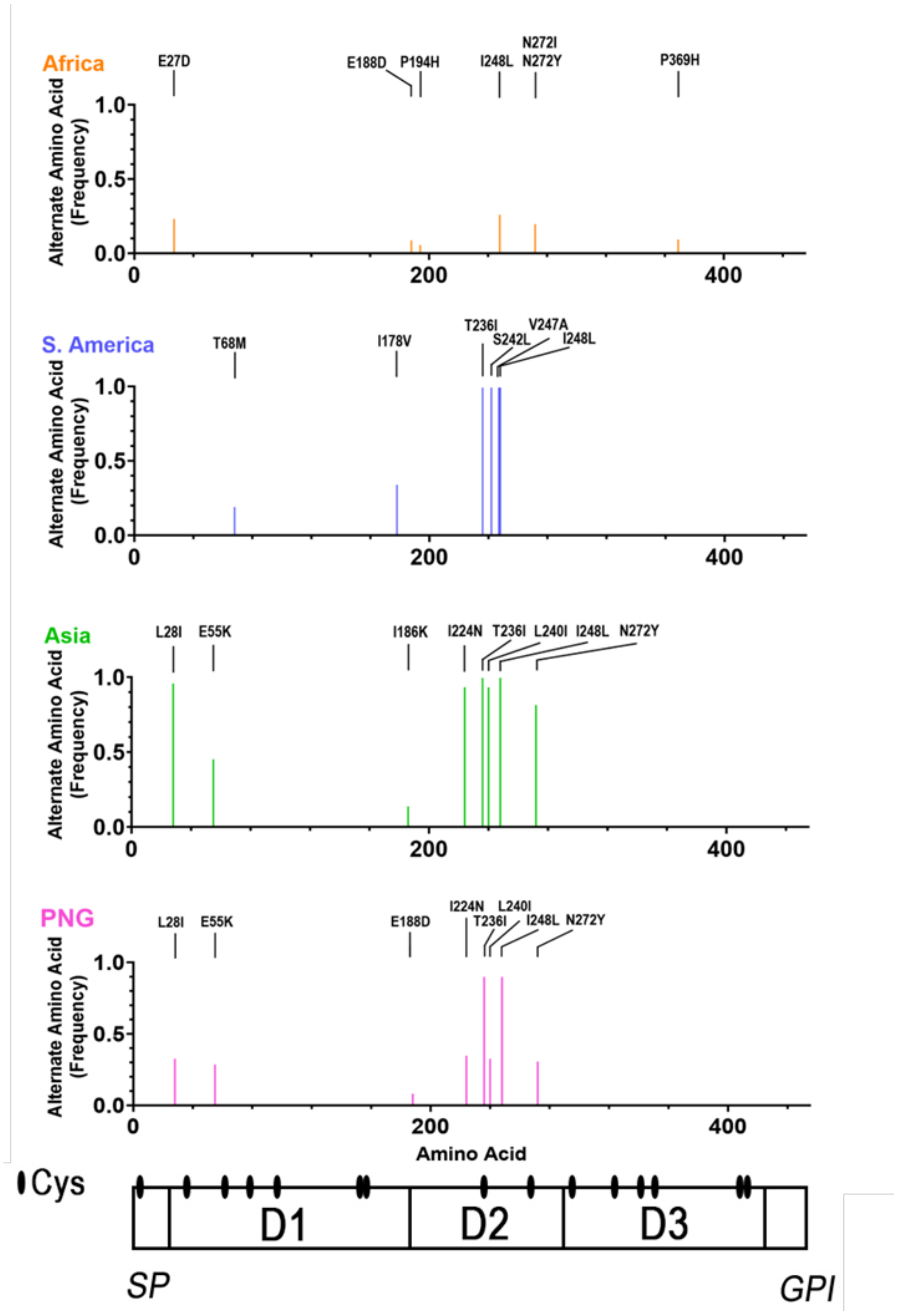

Fig S3. Main amino acid polymorphisms in Pfs47 protein sequences. The alternate amino acid frequencies for polymorphisms that have >0.05 frequency in any of the geographic region analyzed are indicated. The domain structure of Pfs47 is indicated (bottom), with the relative position of Cys. SP, signal peptide; D1, domain 1; D2, domain 2; D3, Domain 3; GPI, predicted glycosilphosphatidyl inositol anchoring; S. America, South America; PNG, Papua New Guinea.

	SP	D1							D2					D3
**Hap /aa**	22	24	27	57	62	82	129	156	194	230	233	262	273	373
Sal-I	F	F	K	S	S	L	V	D	D	V	M	I	I	A
a	L	.	.	.	.	.	.	.	.	.	.	.	.	.
b	L	.	E	.	.	.	.	.	.	.	.	.	.	.
c	L	L	E	.	.	.	.	.	.	.	.	K	.	.
d	L	L	E	.	.	.	.	.	.	.	.	K	.	V
e	L	L	E	.	.	.	.	.	.	.	.	T	M	V
f	L	L	E	.	.	V	.	G	.	.	.	T	M	V
g	L	L	E	.	.	.	.	G	.	.	.	T	M	V
h	L	L	E	.	.	.	.	.	.	.	I	K	.	V
i	L	L	E	.	.	.	.	.	.	I	I	T	.	V
j	L	L	E	.	N	.	.	.	.	I	I	.	V	V
k	L	L	E	T	.	.	.	.	.	.	I	K	.	.
***P.simium*(7)**	L/F	.	K/E	.	.	.	L/V	.	G/D	.	.	.	.	.
***Pv*-like(10)**	L	L	E	S/N	.	.	.	E/D	.	.	.	T	M	.

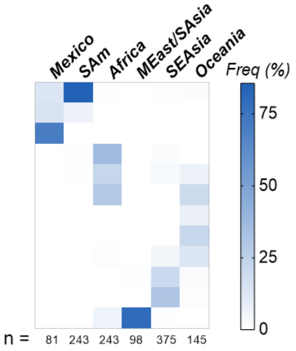

Fig. S4. Frequency of Pv47 aa haplotypes of polymorphisms with strong geographic structure. The Pv47 haplotypes shown (Sal-I, a-k) are defined by SNPs that exhibit a marked population structure (F_{ST}>0.2) between any of the regions compared and have a frequency greater than 5% in at least one of the geographic regions analyzed. Polymorphisms found in 7 sequences of *P. simium* P47 and 10 sequences of *P. vivax-like* P47 in the corresponding loci are also shown. SAm, South America; MEast, Middle East; SAsia, South Asia; SEAsia, Southeast Asia.

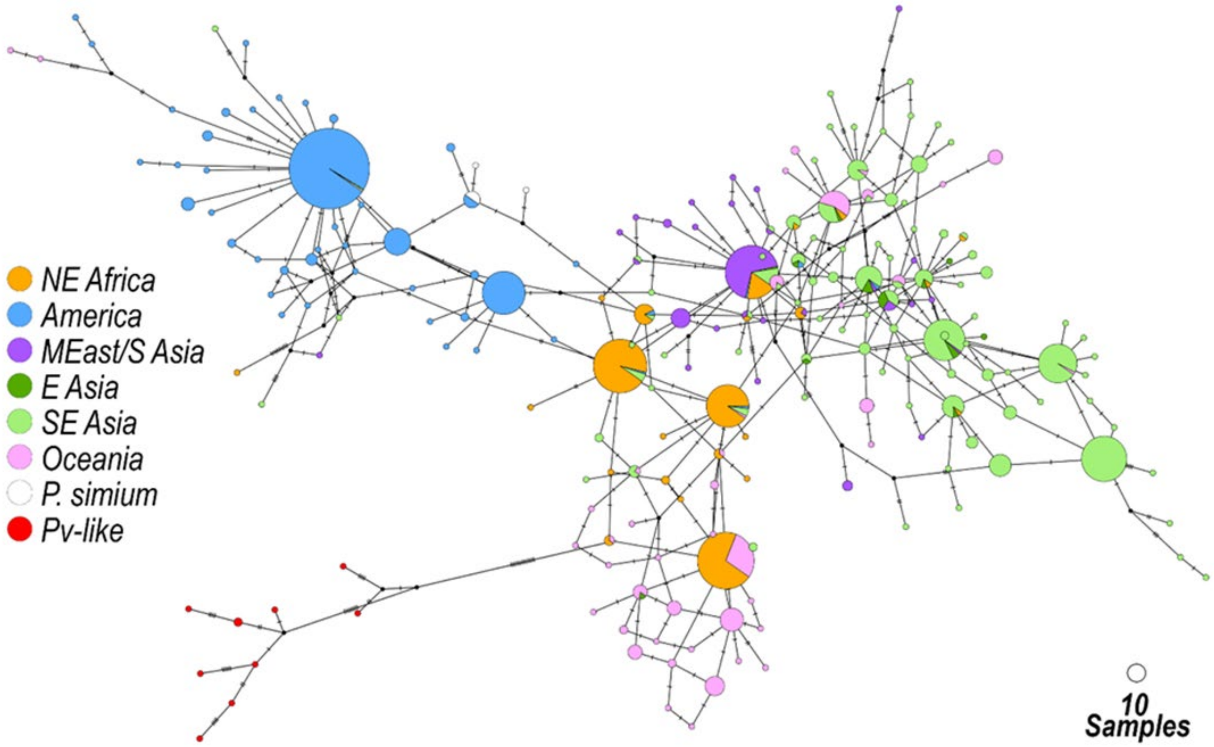

Fig. S5. Genealogy *Pv47*, *P. simium* P47 and *P. vivax*-like P47 haplotypes. Haplotype network (TCS) of the 209 *Pv47* haplotypes identified world-wide. The geographic origin of each haplotype is indicated by a different color. The size of the circular node representing each haplotype is proportional to the number of samples with that sequence (a circle representing 10 samples is shown as reference). The perpendicular marks on the branches between haplotypes indicate the number of nucleotide substitutions separating the two haplotypes. NE Africa, Northeast Africa; America, Mexico and South America; MEast/S Asia, Middle East and South Asia; E Asia, East Asia; SE Asia, Southeast Asia.
