# Supplementary material for: Worldwide genetic diversity of Plasmodium vivax Pv47 is consistent with natural selection by anopheline mosquitoes
Source: Nat Commun. 2025 Aug 9;16:7363. doi: 10.1038/s41467-025-62680-3 (PMC12335571; doi:10.1038/s41467-025-62680-3)
Supplement: Supplementary file 3 — Description of Additional Supplementary Files [file 41467_2025_62680_MOESM3_ESM.pdf]

**Description of Additional Supplementary Files**

File Name: Supplementary Data 1
Description: *Pv47* gene sequences from countries worldwide. A total of 1,191 *Pv47* gene sequences from 28 countries were used to analyzed *Pv47* genetic diversity.

File Name: Supplementary Data 2
Description: *Pv47* gene haplotypes. The 209 *Pv47* haplotypes identified.
